# The Role of Heparanase in the Pathogenesis of Acute Pancreatitis: A Potential Therapeutic Target

**DOI:** 10.1038/s41598-017-00715-6

**Published:** 2017-04-06

**Authors:** Iyad Khamaysi, Preeti Singh, Susan Nasser, Hoda Awad, Yehuda Chowers, Edmond Sabo, Edward Hammond, Ian Gralnek, Irena Minkov, Alessandro Noseda, Neta Ilan, Israel Vlodavsky, Zaid Abassi

**Affiliations:** 1 Department of Gastroenterology, Rambam Health Campus, Haifa, Israel; 2 Cancer and Vascular Biology Research Center, The Ruth & Bruce Rappaport Faculty of Medicine, Technion, Haifa Israel; 3 Department of Physiology, The Ruth & Bruce Rappaport Faculty of Medicine, Technion, Haifa Israel; 4 Pathology, Rambam Health Campus, Haifa, Israel; 5 Zucero Therapeutics, Brisbane, Queensland Australia; 6 HaEmek Medical Center, Afula, Israel; 7 Leadiant Biosciences S.A., Mendrisio, Switzerland; 8 Department of Laboratory Medicine, Rambam Health Campus, Haifa, Israel

## Abstract

Acute pancreatitis (AP) is one of the most common diseases in gastroenterology. However, neither the etiology nor the pathophysiology of the disease is fully understood and no specific or effective treatment has been developed. Heparanase is an endoglycosidase that cleaves heparan sulfate (HS) side chains of HS sulfate proteoglycans into shorter oligosaccharides, activity that is highly implicated in cellular invasion associated with cancer metastasis and inflammation. Given that AP involves a strong inflammatory aspect, we examined whether heparanase plays a role in AP. Here, we provide evidence that pancreatic heparanase expression and activity are significantly increased following cerulein treatment. Moreover, pancreas edema and inflammation, as well as the induction of cytokines and signaling molecules following cerulein treatment were attenuated markedly by heparanase inhibitors, implying that heparanase plays a significant role in AP. Notably, all the above features appear even more pronounced in transgenic mice over expressing heparanase, suggesting that these mice can be utilized as a sensitive model system to reveal the molecular mechanism by which heparanase functions in AP. Heparanase, therefore, emerges as a potential new target in AP, and heparanase inhibitors, now in phase I/II clinical trials in cancer patients, are hoped to prove beneficial also in AP.

## Introduction

Acute pancreatitis (AP) is one of the most common diseases in gastroenterology and about 2% of all hospitalized patients are diagnosed with the disease^1^. The incidence of AP per 100,000 population ranges from 10 to 46 cases per year. However, neither the etiology nor the pathophysiology of the disease are fully understood and thus treatment options targeting a specific underlying cause remain elusive^2, 3^. This has prompted considerable interest into studying the initial triggering events of AP in order to develop novel treatments. Much of our current knowledge regarding AP has been gleaned from animal models or isolated cells of the diseased pancreas^2, 4^. The most common experimental animal model is induction of AP by cholinergic agonists such as carbamylcholine (carbachol), cholecystokinin (CCK) and its analogues, or by scorpion venom^2–4^. Lampel and Kern characterized the clinical and biochemical pattern of acute interstitial pancreatitis in rats after administration of excessive doses of pancreatic secretagogue and thus established the model of secretagogue-induced pancreatitis^5^. The most prominent characteristic is the development of excessive edema as early as one hour after the onset of the disease, and induction of tissue inflammation^2, 4, 6^. Cerulein is a CCK analogue derived from the Australian tree frog *Litoria caerulea* and is one of the best characterized AP models in mice^2, 4, 6^.

Heparanase is an endoglycosidase that acts both at the cell surface and extracellular matrix (ECM) to cleave heparan sulfate (HS) side chains of HS proteoglycans (HSPGs) into shorter oligosaccharides^7, 8^. Heparanase activity is implicated in the regulation of various physiological and pathological processes^9–12^. Cleavage of HS by heparanase facilitates structural alterations of the ECM and thereby promotes cell invasion associated with inflammation, tumor metastasis and angiogenesis^10–14^. Because of its pluripotent pro-inflammatory effects^12^, we hypothesized that heparanase may be involved in the pathogenesis of AP. Here, we provide evidence that pancreatic heparanase expression and activity are significantly increased (over 6-fold) following cerulein treatment. Moreover, pancreas edema and inflammation (i.e., recruitment of neutrophils), as well as the induction of cytokines (i.e., TNFα, IL-6) and signaling molecules (i.e., phospho-STAT3) following cerulein treatment were attenuated markedly by heparanase inhibitors, suggesting that heparanase plays a significant role in AP. We further show that cerulein treatment induces the expression of cathepsin L which is responsible for heparanase processing and activation. Notably, all the above features appear even more pronounced in transgenic mice over expressing heparanase (Hpa-Tg), suggesting that these mice can be utilized as a most sensitive model system to further reveal the molecular mechanism by which heparanase functions in AP. Heparanase, therefore, emerges as a potential new target in AP, and heparanase inhibitors, now in phase I/II clinical trials in cancer patients, may prove beneficial also in AP.

## Results

### Heparanase expression and activity are induced in AP

**Figure 1:**
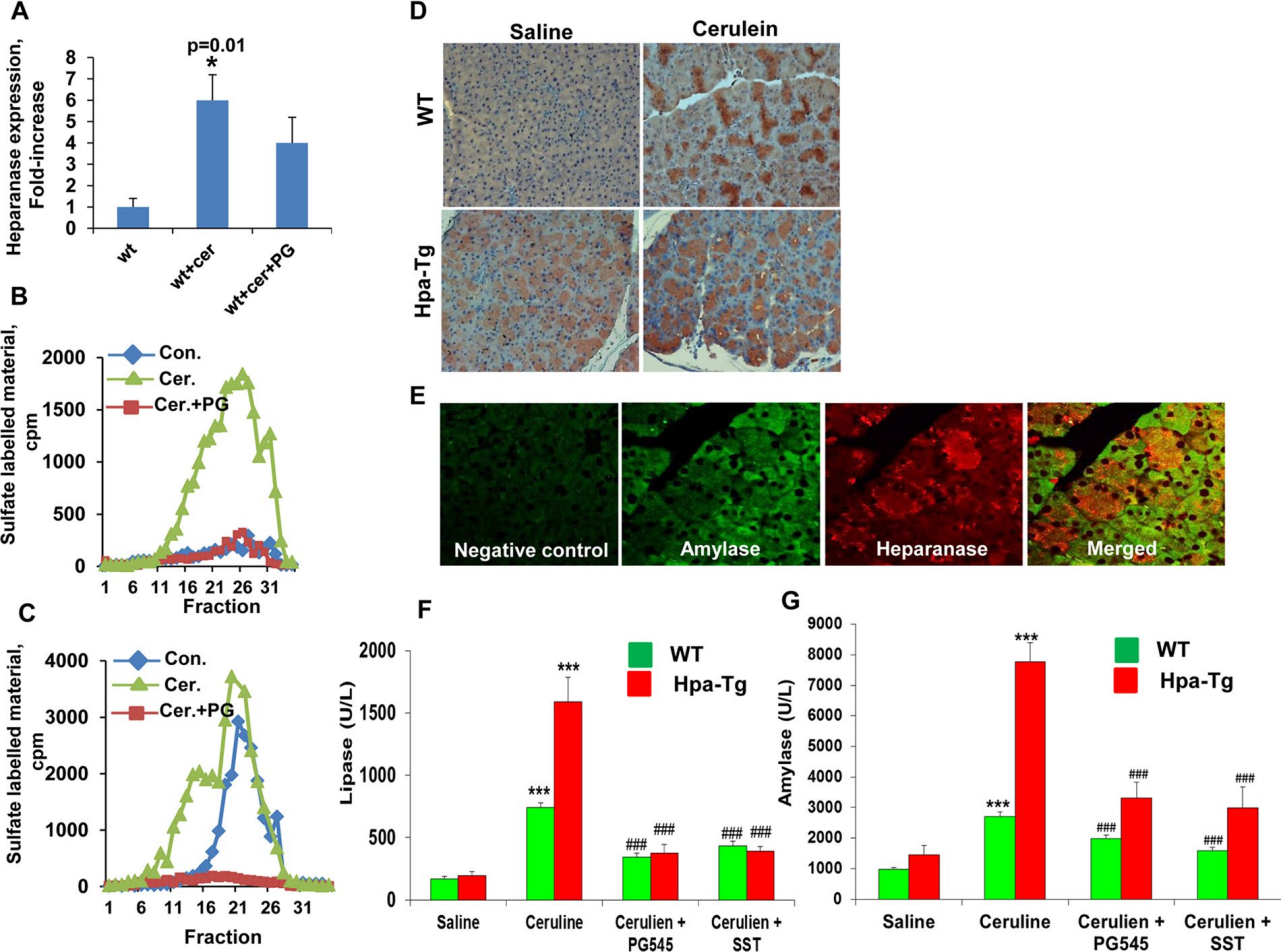
(**A**) Cerulein treatment induces heparanase expression. Pancreas tissue was harvested from control mice (WT) or mice treated with cerulein in the absence (wt+cer) or presence of PG545 (wt+cer+PG). Total RNA was extracted and subjected to quantitative real-time PCR applying heparanase primers. Relative heparanase gene expression (fold-increase) is shown graphically in relation to heparanase levels in control pancreas set arbitrarily to a value of 1. Heparanase mRNA levels were normalized to actin mRNA (number of mice in each group = 5); *p < 0.01 for control vs. cerulein. Heparanase enzymatic activity was evaluated in corresponding control (blue), cerulein (green) and cerulean+PG545 (red) pancreatic tissue extracts (**B**). Note most noticeable increase in heparanase activity following cerulein treatment, which is almost completely inhibited by PG545. (**C**) Heparanase activity was similarly evaluated in pancreatic tissues harvested from Hpa-Tg (blue), Hpa-Tg+ cerulein (green) and Hap-Tg+ cerulean+PG545 (red). (**D,E**) Immunostaining. Control untreated (Saline) and cerulein-treated pancreas tissues were harvested from WT (upper panels) and Hpa-Tg (lower panels) mice, fixed with formalin and embedded in paraffin. 5-micron sections were subjected to immunostaining applying anti-heparanase antibody (**D**). (**E**) Corresponding sections of cerulein-treated WT mice were subjected to immunofluorescence staining applying anti-amylase (green, second left panel) and anti-heparanase (red, second right panel) antibodies. Merged image is shown in the right panel. Background staining, omitting the primary antibody, is shown in the left panel (Negative control). Note co-localization (orange-yellow) of amylase and heparanase in acinar cells. Shown are representative images at x20 original magnification. (**F,G**) Induction of lipase and amylase by cerulein is restored by heparanase inhibitors. Blood samples were collected from control untreated WT (n = 13) or Hpa-Tg (n = 11) mice (saline) or mice treated with cerulein in the absence (cerulein; n = 24) or presence of PG545 (cerulean+PG545; n = 20) or SST0001 (cerulean+SST; n = 6) and evaluated biochemically for lipase (**F**) and amylase (**G**) levels. ***P < 001 for saline vs. cerulein; ^###^P < 001 for cerulein vs. cerulean+PG545/SST. Note, reduced levels of lipase and amylase in mice treated with the heparanase inhibitors.

In order to reveal the significance of heparanase in pancreatitis, we applied a well-established cerulein-based mouse model. Notably, heparanase mRNA (Fig. 1A) and activity (Fig. 1B, green) levels were significantly elevated (over 6-fold) in cerulein-treated wild-type (WT) pancreas. Importantly, the high heparanase activity observed following cerulein treatment was abrogated by the heparanase inhibitor PG545 (Fig. 1B, red) and Roneparstat (formerly SST0001; Suppl. Fig. 1A, red), whereas heparanase expression was not significantly affected by PG545 (Fig. 1A, +PG). Increased heparanase levels were similarly observed by immunostaining of cerulein-treated WT pancreas (Fig. 1D, upper panels), and double immunofluorescent staining for heparanase (red) and amylase (green) revealed that exocrine pancreatic cells are the heparanase-producing cells (Fig. 1E, right panel; yellow-orange). Serum levels of amylase and lipase typical of AP were increased substantially (3–5 fold) in cerulein-treated WT mice (Fig. 1F,G; green) and even higher ~7 fold induction of lipase and amylase levels was noted in Hpa-Tg mice following cerulein treatment (Fig. 1F,G; red bars), associating with higher levels of heparanase in Hpa-Tg pancreas (Fig. 1C and D, = SST). Notably, this elevation was markedly reduced by the heparanase inhibitors PG545 and Roneparstat in WT and Hpa-Tg mice (Fig. 1F,G) altogether implying that heparanase plays a substantial role in AP.

### Histological and ultrastructural analyses

**Figure 2:**
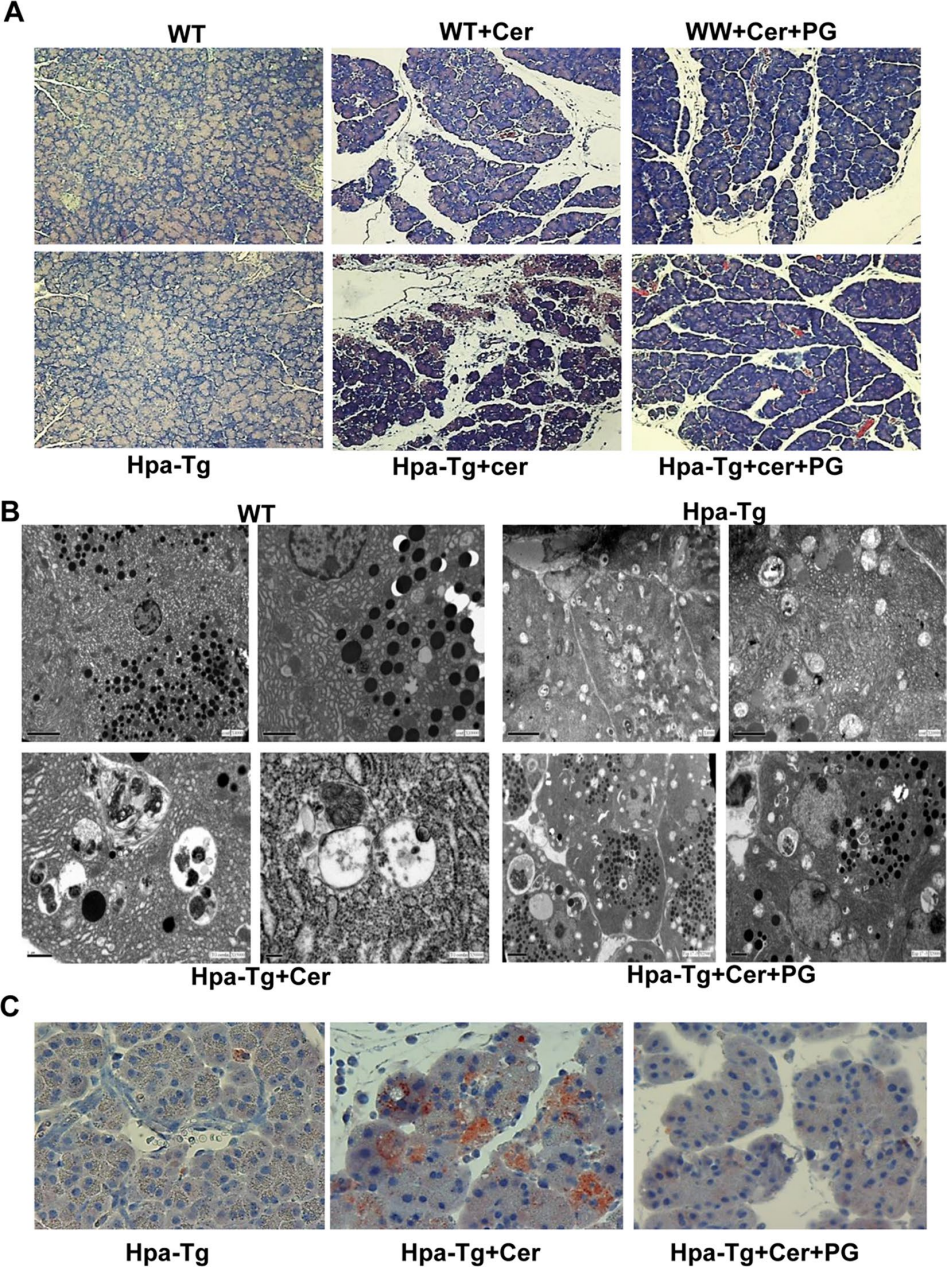
Histological analyses. (**A**) H&E staining. WT and Hpa-Tg mice were injected with saline (left panels) or cerulein in the absence (middle panels) or presence (right panels) of PG545 pretreatment. Pancreas tissues were collected 24 h thereafter, and 5 micron sections from formalin-fixed, paraffin-embedded samples were stained for H&E (**A**). Shown are representative photomicrographs at 10x original magnification. Corresponding pancreas tissues were processed for electron microscopy as described under ‘Materials and Methods’ (**B**). Note, increased autophagy in pancreatic acinar cells of Hpa-Tg vs. WT mice; Autophagy is further enhanced by cerulein, but restored by PG545 (cerulein+PG). Induction of AP resulted in dilatation of RER, mitochondrial swelling and large autophagosomes. Pancreatic tissue from mice treated with PG545 exhibited nearly normal ultrastructural appearance. Shown are representative micrographs at x8000, x10,000 and x12,500 original magnification. (**C**) LC-3 immunostaining. Hpa-Tg mice were injected with saline (left) or cerulein in the absence (middle) or presence (right) of PG545 pretreatment. Pancreas tissues were collected 24 h thereafter, and 5 micron sections from formalin-fixed, paraffin-embedded samples were stained for LC-3 (marker of autophagy). Shown are representative photomicrographs at original magnification of x40. Note that LC-3 staining resembles the patchy pattern of heparanase staining (Fig. 1D,E).

To examine further the involvement of heparanase in the pathogenesis of AP, pancreatic morphology was evaluated in WT and Hpa-Tg mice by histopathological and electron microscopy analyses. Cerulein treatment resulted in typical edema in WT mice and even more sever edema was noted in Hpa-Tg mice exposed to cerulein (Fig. 2A, left vs. middle panels). Notably, tissue edema was markedly restored in mice treated with PG545 (Fig. 2A, middle vs. right panels), further supporting the notion that heparanase plays a role in AP. Electron microscopy revealed that Hpa-Tg pancreas cells are decorated with increased number of cytoplasmic vacuoles typical of autophagosomes (Fig. 2B, upper panels), and the vacuole size was increased substantially following cerulein treatment (Fig. 2B, left lower panels), suggesting that cerulein enhances zymophagy and autophagy. Indeed, the levels of LC3, a most commonly used marker of autophagy, was increased following cerulein treatment (Fig. 2C). Importantly, number and size of autophagosomes and levels of LC3 were noticeably decreased by PG545 (Fig. 2B,C, right panels), suggesting that heparanase functions to promote autophagy in AP, as noted previously in cancer cells^15^. In fact, PG545 abolished the deleterious ultrastructural alterations induced by cerulein, yielding almost normal appearance of the rough endoplasmic reticulum, Golgi apparatus and mitochondria (Fig. 2B).

### Heparanase enhances neutrophil infiltration and induces NFκB and STAT3 activation

**Figure 3:**
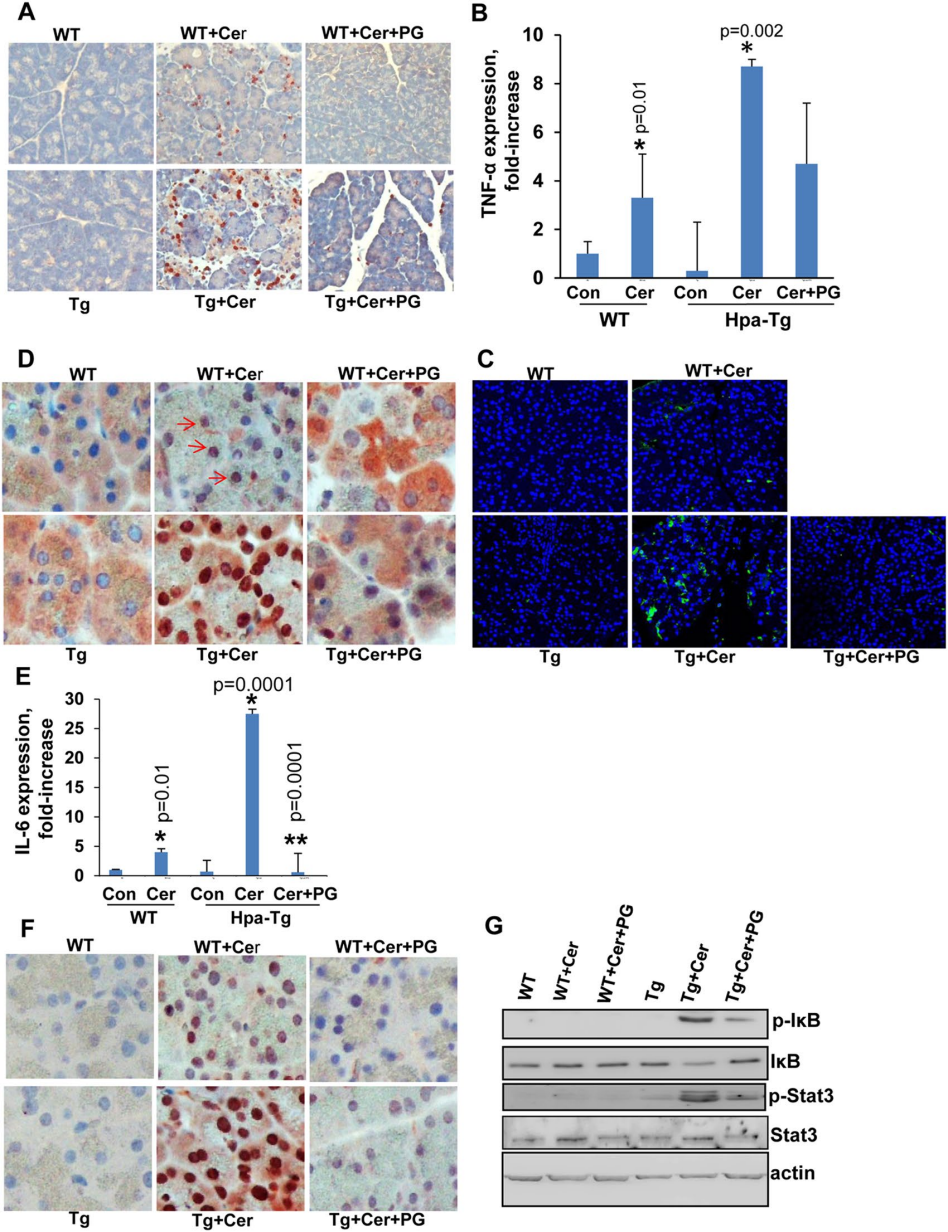
Pancreas inflammation, cytokine induction and signaling pathways evoked by cerulein are attenuated by heparanase inhibitor. WT and Hpa-Tg mice were injected with saline (left panels) or cerulein in the absence (middle panels) or presence (right panels) of PG545 pretreatment. Pancreas tissues were collected 24 h thereafter, and 5 micron sections from formalin-fixed, paraffin-embedded samples were stained for neutrophils infiltration (**A**), p65 (**D**) and phospho-STAT3 (**F**). Immunofluorescent staining of corresponding sections for TNFα (green) is shown in (**C**) along with nuclear counterstaining (blue). Shown are representative photomicrographs at x10 (**A**) and x40 (**C,D,F**) original magnification. Total RNA was extracted from corresponding pancreas tissues and subjected to real-time PCR analyses applying primers specific for TNFα (**B**) and IL-6 (**E**). Relative gene expression (fold-increase) is shown graphically in relation to the levels in control pancreas set arbitrarily to a value of 1. *p < 0.01 for control vs. cerulein; **p < 0.001 for cerulein vs. cerulein+PG545. (**G**) Immunoblotting. Protein extracts from corresponding pancreas tissues were subjected to immunoblotting applying anti-phospho-IκB (upper panel), anti-IκB (second panel), anti-phospho-STAT3 (third panel), anti-STAT3 (fourth panel) and anti-actin (lower panel) antibodies.

Pathological examination revealed that in addition to increased edema, cerulein treatment also enhances pancreatic inflammation (Fig. 2A, middle panels). More specifically, we found that cerulein treatment resulted in the recruitment of neutrophils evident in WT mice (Fig. 3A, upper panels) and even more so in Hpa-Tg mice (Fig. 3A, lower panels). Similar to the biochemical determinants (i.e., lipase and amylase; Fig. 1F,G), the recruitment of neutrophils was attenuated markedly by PG545 in WT and Hpa-Tg mice (Fig. 3A, right panels). Neutrophil recruitment is a central characteristic feature of AP^2, 4, 6, 16^. Activated neutrophils in AP secrete various cytokines including TNFα, a key cytokine implicated in experimental and clinical pancreatic injury^17^. Indeed, TNFα expression was elevated 3-folds in WT pancreas following cerulein treatment and even higher, 9-fold induction of TNFα expression was found in cerulein-treated Hpa-Tg pancreas (Fig. 3B; Hpa-Tg). This was evident by quantitative real-time PCR analysis (Fig. 3B) and immunofluorescent staining (Fig. 3C, green). The induction of TNFα was associated with a substantial increase in IκB phosphorylation (Fig. 3G, upper panel) and a concomitantly accumulation of p65 in the cell nuclei (Fig. 3D), strongly implying activation of the NFκB signaling pathway. Notably, induction of TNFα, increased IκB phosphorylation and nuclear translocation of p65 were all prominently decreased by PG545 (Fig. 3B; +PG). A similar expression pattern was noted for IL-6 (Fig. 3E), associating with a significant increase in STAT3 phosphorylation (Fig. 3F and G, third panel), a typical downstream effector of IL-6, that was considerably decreased by PG545 (Fig. 3F, right panels; Fig. 3G, third panel, +pg) and Roneparstat (Supp. Fig. 1B). Thus, heparanase induction by cerulein is associated with activation of key signaling pathways that function to promote AP, whereas heparanase inhibitors efficiently restore the damage.

### Heparanase enhances cathepsin-L expression in the course of cerulein-induced AP

**Figure 4:**
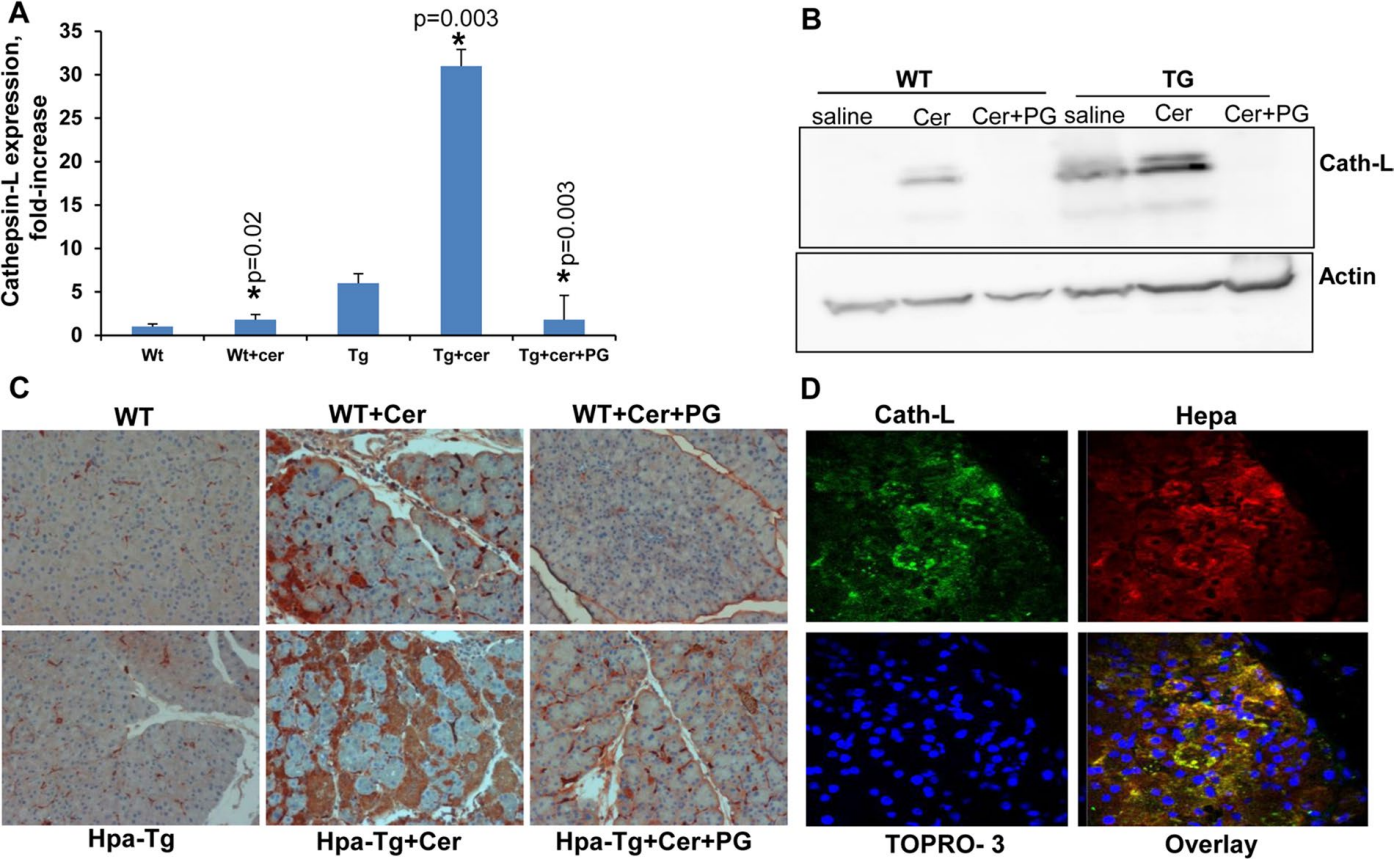
Cerulein induces the expression of cathepsin-L in a heparanase-dependent manner. (**A**) Real-time PCR. WT and Hpa-Tg mice were injected with saline or cerulein in the absence or presence of PG545 pretreatment. Total RNA was extracted from pancreas tissues 24 hours thereafter and subjected to real-time PCR applying primers specific for cathepsin L. Relative gene expression (fold-increase) is shown graphically in relation to the levels in control pancreas set arbitrarily to a value of 1. (**B**) Immunoblotting. Proteins were extracted from corresponding pancreas tissues and lysate samples were subjected to immunoblotting applying anti-cathepsin L (upper panel) and anti-actin (lower panel) antibodies. (**C**) Immunostaining. Corresponding paraffin embedded pancreatic tissue sections were subjected to immunostaining applying anti- cathepsin-L antibody. Note a striking increase in cathepsin L expression by cerulein which is restored by the heparanase inhibitor PG545. (**D**) Colocalization of cathepsin L and heparanase. Paraffin embedded, pancreatic tissue sections of cerulein treated Hpa-Tg mice were subjected to double immunofluorescence staining using antibodies against cathepsin L (green) and heparanase (red). Merged image is shown together with nuclei counterstaining (blue). Co-localization appears in yellow. Shown are representative photomicrographs at original magnification x40.

The heparanase gene encodes a latent 65 kDa proenzyme whose activation involves proteolytic cleavage by cathepsin L (CatL), yielding an enzymatically-active heterodimer composed of 8- and 50-kDa subunits^18–20^. Interestingly, we found that CatL expression is induced in WT mice treated with cerulein. This was evident by quantitative real-time PCR (Fig. 4A), immunoblotting (Fig. 4B) and immunostaining (Fig. 4C). Double immunofluorescent staining for CatL (green) and heparanase (red) showed that the two proteins co-localize in apparently infiltrating cells or centroacinar cells with a swollen duct (Fig. 4D; yellow). Even higher induction of CatL expression was noted in Hpa-Tg mice treated with cerulein (Fig. 4A–C; Tg), and this induction was attenuated noticeably by PG545 (Fig. 4A–C; +PG). Thus, the induction of heparanase expression in the course of AP is accompanied by enhanced expression of CatL which, in turn, activates heparanase in a loop that feeds itself, generating continuous production of active heparanase enzyme that apparently functions to support AP. This devastating loop is efficiently blocked, nonetheless, by the heparanase inhibitors PG545 and Roneparstat, lending hope that these compounds, now in phase I/II clinical trials in cancer patients will prove efficacious also in AP.

## Discussion

Acute pancreatitis is mild in most cases and without serious complications. Nevertheless, it is associated with morbidity and mortality in up to 20% of patients^2, 6, 16^. AP has been intensively investigated for decades, yet neither the etiology nor the pathophysiology of the pancreatitis process are fully understood and no specific or effective treatment has been developed^2, 16, 21^. Here, we provide for the first time evidence that heparanase is engaged in AP. This is concluded for the following considerations. Heparanase expression and activity were noticeably increased by cerulein (Fig. 1A,B,D). This is in agreement with induction of heparanase expression, to a comparable extent, in human chronic pancreatitis^22^, signifying a clinical relevance of our mouse model. Notably, all the key determinants associating with cerulein-induced AP, namely induction of lipase and amylase (Fig. 1F,G), increased tissue edema (Fig. 2A), recruitment of neutrophils (Fig. 3A), induction of cytokines (i.e., TNFα, IL-6), activation of NFκB and STAT3 signaling (Fig. 3B–G; Suppl. Fig. 1B) as well the pancreatic index (Suppl. Fig. 1C) were attenuated significantly by the heparanase inhibitors PG545 and Roneparstat. Inhibition of lipase and amylase induction by cerulein were attenuated significantly even when PG545 was administrated at the onset of pancreatitis induction (Suppl. Fig. 1E,F), further signifying the potency of this compound. The consistent performance of two different inhibitory molecules strongly implies that heparanase plays an important role in the course of AP. In favor of this notion is the observation that all the above parameters were even more prominent in Hpa-Tg mice endowed with higher levels of heparanase in their pancreas (Fig. 1C,D)^23^, suggesting that the severity of AP correlates with heparanase levels.

In line with this concept, we found that heparanase knockout (Hpa-KO) mice show increased lipase levels in response to cerulein, albeit to a lower extent than those seen in WT mice (not shown). However, we have previously reported that the Hpa-KO mice compensate for the lack of heparanase by up-regulating the expression of matrix degrading enzymes (primarily MMP14 and MMP2)^24^ resulting in, paradoxically, several phenotypes (i.e., wound angiogenesis, branching morphogenesis) that closely resemble those seen in Hpa-Tg mice^24^. This compensatory mechanism makes Hpa-KO mice inappropriate model system in some cases, including the cerulein-AP model.

While our study implicates heparanase with AP, it should be envisioned in a broad perspective that ties heparanase with fundamental function of the pancreas. More specifically, it has recently been shown that HS is essential for the survival of pancreatic beta cells; *In vivo*, autoimmune destruction of islets was associated with production of catalytically active heparanase by islet-infiltrating mononuclear cells, and loss of islet HS^25, 26^. Furthermore, treatment with a heparanase inhibitor preserved intra islet HS and protected mice from type I diabetes^25, 26^. Thus, heparanase inhibitors may turn even more important for chronic pancreatitis, protecting beta cell islets from destructive heparanase produced by pancreatic acinar cells (Fig. 1E) and/or inflammatory cells.

The cerulein model is widely used for exploring the mechanisms underlying the pathogenesis of AP and for testing novel therapeutic strategies^3, 21, 27^. As expected, cerulein-induced pancreatitis is characterized by elevated levels of amylase and lipase along with extensive inflammation as evident by infiltration of neutrophils (Fig. 3A) and pancreatic edema (Fig. 2A; Suppl. Fig. 1C). It is generally thought that these morphologic changes result from digestion of the pancreas by enzymes that are normally synthesized and secreted by pancreatic acinar cells as zymogens^2, 4, 6, 16^. Here, we found that cathepsin L is another enzyme induced by cerulein (Fig. 4) and likely contributes to AP. This notion is supported by publications showing that the severity of pancreatitis is reduced in cathepsin L-KO mice^28^. In this context, cathepsin L functions not only to damage the pancreas by its digestive activity, but also to activate heparanase. Heparanase is first synthesized as a readily secreted inactive 65 kDa pro-enzyme that is taken up by the cells and transferred to endosomes/lysosomes^29, 30^. In the lysosomes, heparanase is proteolytically processed by cathepsin L into its active form, a heterodimer constituted of 8 kDa and 50 kDa subunits^18, 20, 31^. In addition, latent heparanase can get activated extracellularly by secreted cathepsin L. For example, in experimental dextran sodium sulfate (DSS)-induced chronic colitis in mice it was found that activated macrophages secretes active cathepsin L which in turn activates latent heparanase that is secreted by colon epithelial cells^32^. In pancreatitis, heparanase is expressed by pancreatic acinar cells (Fig. 1E) and infiltrated cells or possibly centroacinar cells, in which heparanase partially co-localizes with cathepsin L (Fig. 4D, overlay, yellow).

The continuous presence of active heparanase extracellularly can profoundly promote inflammation thought to play an important role in AP. HS is known to control inflammatory responses at multiple levels, including sequestration of cytokines/chemokines in the ECM, modulation of leukocyte interactions with endothelium and ECM, and initiation of innate immune responses through interactions with toll-like receptor 4 (TLR4)^33–40^. Thus, HS remodeling by heparanase may affect several aspects of inflammatory reactions, such as leukocyte recruitment, extravasation and migration towards inflammation sites; release of cytokines and chemokines anchored within the ECM or cell surfaces, as well as activation of innate immune cells^9, 41, 42^. Mounting evidence suggests that heparanase affects activities of several types of innate immunocytes, including neutrophils, macrophages, dendritic and mast cells^32, 43–48^. Of those, neutrophils represent the important effectors in acute inflammatory responses, including AP. In a mouse model of sepsis-associated inflammatory lung disease, rapid induction of heparanase activity was demonstrated in pulmonary microvascular endothelial cells^47^. This was associated with degradation of the glycocalyx, a thin gel-like layer that coats the luminal surface of blood vessels, leading to increased availability of endothelial surface adhesion molecules and consequently, improved neutrophil adhesion and extravasation^47^. Heparanase inhibition prevented endotoxemia-associated glycocalyx loss and neutrophil adhesion and, accordingly, attenuated sepsis-induced acute lung injury and mortality in mice. Likewise, reduced infiltration of neutrophils and eosinophils was noted in Hpa-KO lungs exposed to prolonged smoke exposure or subjected to allergic inflammatory model^49, 50^. Reduced neutrophils recruitment in mice administrated with PG545 prior to cerulein treatment (Fig. 3A) is in agreement with anti-inflammatory effects demonstrated for heparanase-inhibiting substances (i.e., heparin, heparin-mimicking compounds) in animal and clinical studies^9, 51–55^ further supporting involvement of the enzyme in inflammatory reactions. Likewise, ample recruitment of neutrophils to Hpa-Tg vs. WT pancreas in response to cerulein (Fig. 3A) further ties heparanase levels with the amplitude of inflammatory reactions. This is also reflected by augmented levels of TNFα, IL-6 and NFκB in Hpa-Tg vs. WT pancreas, signaling determinants that are central to AP^4^. Notably, heparanase enhances the IL-6/p-STAT3 axis also in inflammation (macrophages)-driven colon^32^ and pancreatic^56^ cancer progression, suggesting that the principle function of heparanase is preserved in different immunocytes.

Heparanase is also strongly implicated in activation of macrophages that take central stage when acute inflammation is not properly resolved and turn into chronic phase. Heparanase has been shown to strongly augment activation of macrophages, resulting in increased production of pro-inflammatory cytokines (i.e., TNFα, MCP-1, IL-6, IL-1β)^32, 45, 56, 57^, whereas decreased cytokine levels were quantified in macrophages isolated from Hpa-KO mice^58^. Mechanistically, we have recently identified a linear cascade that starts with heparanase-mediated activation of TLRs at the cell membrane, continues with Erk/p38/NFκB/JNK activation, leading to increased c-Fos levels and induction of cytokine expression^58^, but it relevance to AP awaits further investigation.

It should be mentioned that along with pro-inflammatory cytokines (IL-6 and TNFα), cerulein also enhanced the expression of seemingly anti-inflammatory (IL-10) cytokines (Suppl. Fig. 1D), in a manner that involves heparanase because this induction was significantly decreased by PG545 (Fig. 3). Regulation of IL-10 expression by heparanase was observed in other systems^56–58^, suggesting that inflammation and regeneration are simultaneously ongoing in this model of AP. It should be kept in mind, though, that classification into pro-inflammatory and anti-inflammatory cytokines is far too simplistic. In fact, the cytokine amount, the nature of the target cell, the nature of the activating signal, the timing and the sequence of cytokine action will dictate if a given cytokine will behave as a pro- or anti-inflammatory cytokine^59^. For example, a pro-inflammatory effect of IL-10 has been reported in some cases^60, 61^. Given its pluripotent effects, heparanase appears to exert both pro-inflammatory and anti-inflammatory effects, depending on the context. This is best exemplified in studies showing that neuro-inflammation is inhibited in response to heparanase^62, 63^. The inhibitory effect is likely due to disruption of chemokine gradients and the resulting impaired extravasation of blood-born immune cells upon degradation of HS on the surface of vascular endothelial cells^46^.

Accumulation of large vacuoles in acinar cells is a long-noted and prominent feature of experimental and human pancreatitis^64^. Subsequent studies identified these vacuoles as autophagosomes^64^, but the role of autophagy during pancreatitis has been controversial^21^. Genetic inhibition of autophagy reduced trypsinogen activation and pancreatic damage indicating that autophagy promotes AP^65^. In contrast, other reports described a selective autophagy which is protective during early pancreatitis response^66^. We have reported previously that autophagy (i.e., LC-3II levels) is increased substantially in the pancreas of Hpa-Tg vs. WT mice^15^, and this increase is further confirmed here by EM analysis (Fig. 2B, upper panels). Notably, autophagy is further increased following cerulein treatment (Fig. 2B, left panels), but is restored to base line levels by PG545 (Fig. 2B, right panels). Thus, in our model system, autophagy levels correlate with heparanase levels and the severity of AP, but more intense research is required to reveal the mechanism responsible for autophagy regulation by heparanase in the course of AP.

Taken together, to the best of our knowledge, this is the first study to demonstrate that heparanase is involved in the pathogenesis of AP. Apparently, the ability of the heparanase inhibitors PG545 and Roneparstat to attenuate cerulein-induced AP appears superior compared with other compounds tested in this experimental model, lending optimism that these heparanase-inhibiting compounds will prove efficacious in the clinic for AP and other heparanase-driven diseases.

## Materials and Methods

### Animals

Studies utilized wild type (WT) BALB/c mice (n = 13–24) and heparanase transgenic (Hpa-Tg) mice (n = 10–22) in which the human heparanase gene is driven by a constitutive β-actin promoter^23^ in a BALB/c genetic background. Animals were fed standard mouse chow and tap water ad libitum. All animal experiments were performed according to the Guide for the Care and Use of Laboratory Animals (NIH Publication No. 85-23) and approved by the Technion (Israel Institute of Technology) committee for the supervision of animal experiments (approval #IL-094–07–013). The Technion is certified by the NIH to run experiments using live animals and the animal facility of the Technion’s Faculty of Medicine has been given the animal welfare Assurance Identification Number: OPRR-A5026-01.

### Induction of acute pancreatitis

Mice were injected with either cerulein (intraperitoneally, 50 µg/kg, 5 times at 1 hour apart) (Sigma-Aldrich), or saline (0.9% NaCl) (control group)^2^. Additional groups of mice were pretreated with PG545 (0.4 mg/mouse, i.p, 2 or 24 h prior to cerulein administration) or Roneparstat (1.0 mg/mouse, i.p, 30 min and 24 h prior to the administration of cerulein). PG545 and Roneparstat were kindly provided by Zucero Therapeutics (Brisbane, Australia) and Leadiant Biosciences (Rome, Italy), respectively^67, 68^. Mice were sacrificed 24 hours later and serum samples and pancreatic tissue were collected for measurements of blood amylase and lipase levels, pancreatic index (pancreas/body weight ratio), and for histological analysis. Portion of pancreas tissues were also homogenized and lysate samples were subjected to immunoblotting.

### Pancreatic histopathology and ultrastructural analyses

#### Light microscopy

Pancreatic tissue samples were fixed in 10% neutral-buffered formalin progressively dehydrated in graduated alcohol and embedded in paraffin. Five micron sections were stained with hematoxylin and eosin (H&E).

#### Electron Microscopy

Pancreatic tissues from the various experimental groups were fixed in 3.5% glutaraldehyde and rinsed in 0.1 M sodium cacodylate buffer, pH 7.4. Tissue blocks (1 mm^3^) were post-fixed with 2% OsO_4_ in 0.2 M cacodylate buffer for 1 h, rinsed again in cacodylate buffer to remove excess osmium, immersed in saturated aqueous uranyl acetate, dehydrated in graded alcohol solutions, immersed in propylene oxide, and embedded in Epon 812. Ultrathin sections (80 nm) were mounted on 300-mesh, thin-bar copper grid, counterstained with saturated uranyl acetate and lead citrate. Sections were examined with a transmission electron microscope (Jeol 1011 JEM), at 80 KV.

### Immunohistochemistry

Pancreatic tissue samples were fixed in 4% PFA and embedded in paraffin. 5 μm-thick sections were deparaffinized, rehydrated and endogenous peroxidase activity was quenched (30 min) by 3% hydrogen peroxide in methanol. Slides were then subjected to antigen retrieval by boiling (20 min) in 10 mM citrate buffer, pH 6. Slides were incubated with 10% normal goat serum (NGS) in phosphate buffered saline (PBS) for 60 min to block nonspecific binding and incubated overnight at 4 °C with antibodies directed against LC-3 (Cell signaling #2775), cathepsin L (1:200, R&D systems, #AF1515), heparanase (1:150, #733 raised against the N terminus of the 50 kDa heparanase subunit)^20^, p65 NFκB subunit (1:100, Santa Cruz Biotechnology, sc-372), and pSTAT3 (1:100, Santa Cruz Biotechnology, sc-8059) diluted in blocking solution. For neutrophil staining sections were incubated with antibody directed against NIMPR 14 (ab2557, Abcam) followed by rat biotin and HRP conjugated streptavidin. Slides were then extensively washed with PBS and incubated with a secondary reagent (Envision kit) according to the manufacturer’s (Dako, Glostrup, Denmark) instructions. Following additional washes, color was developed with the AEC reagent (Dako), sections were counterstained with hematoxylin and mounted as described previously^58, 69, 70^. Images were acquired by a Nikon ECLIPSE microscope and Digital Sight Camera (Nikon, NY, USA). For immunofluorescence analysis, sections were incubated with anti-TNFα (Abcam ab6671), anti-heparanase, anti-amylase (sc-166349), or anti cathepsin L antibodies. Alexa Fluor® 488 anti-rabbit IgG, Alexa Fluor® 488 anti-Goat IgG and Alexa Fluor® 594 anti-rabbit IgG were used as secondary antibodies (Jackson Laboratories PA, USA). Nuclear staining was performed with TOPRO-3. Images were captured using a Zeiss LSM 700 Confocal microscope and analyzed with Zen software (Carl Zeiss).

### Immunoblotting

Pancreatic tissue samples from the various experimental groups were homogenized on ice with RIPA buffer (150 mM NaCl, 1% NP40, 50 mM Tris pH 8.0, 0.5% sodium deoxycholate and 0.1% SDS) supplemented with a cocktail of protease inhibitors (Roche); The homogenized tissue was then centrifuged and the cleared supernatant was subjected to immunoblotting applying anti-cathepsin L (1:1000, Santacruz biotechnology, sc-6498), anti-pSTAT3 (Santacruz biotechnology, Sc-8059), anti-STAT3 (1:1000, Santa Cruz biotechnology, Sc-7179), anti-pIκB (1:1000, Cell signaling Technology, #2859), anti-IκB (1:1000, Cell signaling Technology, #4812) or anti-actin (1:10000, Sigma Aldrich, A1978) antibodies, essentially as described^58, 69, 70^.

### Quantitative real-time PCR

Total RNA was extracted with TRIzol (Sigma) and RNA (1 µg) was amplified using one step PCR amplification kit, according to the manufacturer’s (ABgene, Epsom, UK) instructions. The following moue primer sets were used:

Cat L F: 5′-ATCAAACCTTTAGTGCAGAGTGG -3′, R: 5′-CTGTATTCCCCGTTGTGTAGC -3′;

β-actin F: 5′-ATGCTCCCCGGGCTGTAT-3′, R: 5′-CATAGGAGTCCTTCTGACCCATTC-3′;

TNFα F: 5′-CCCTCACACTCAGATCATCTTCT-3′, R: 5′-TTGGTCCTTAGCCACTCCTTC -3′;

IL-6 F: 5′-CTGCAAGAGACTTCCATCCAG-3′, R: 5′-AGTGGTATAGACAGGTCTGTTGG-3′;

IL-10 F: 5′-GCTCTTACTGACTGGCATGAG-3′, R: 5′- CGCAGCTCTAGGAGCATGTG-3′; heparanase F: 5′-GGGGTTCGTAGTAACGCATTTAG-3′, R: 5′-GCACCTACTCAAGAAGCTCAG-3′.

### Heparanase Activity

Preparation of sulfate-labeled ECM-coated dishes and determination of heparanase activity were performed essentially as described previously^71^. Briefly, freshly collected pancreatic tissues were homogenized and extracted in phosphate/citrate buffer (pH 5.2) by three cycles of freeze/thaw. Tissue lysates were incubated (overnight, 37 °C) with ^35^S-labeled ECM. The incubation medium (1 ml) containing sulfate-labeled degradation fragments were subjected to gel filtration on a Sepharose CL-6B column. Fractions (0.2 ml) were eluted with PBS and their radioactivity counted in a β-scintillation counter. Degradation fragments of HS side chains produced by heparanase are eluted at 0.5 < Kav < 0.8 (fractions 12–29). Nearly intact HSPGs released from the ECM are eluted just after the Vo (Kav < 0.2, fractions 3–10)^24, 45, 58^. These high molecular weight products are released by proteases that cleave the HSPG core protein.

### Statistical analysis

Data are presented as mean of repeated measurements ± standard error (S.E.M). Comparison between two parametric groups was performed using the unpaired Student t test after testing for equality of variances. More than two paired groups were tested using the one-way analysis of variance (ANOVA) test for repeated measurements, followed by the Bonferroni post-hoc test for multiple comparisons.

## Electronic supplementary material

Supplementary Materials
