# Supplementary material for: The Role of Heparanase in the Pathogenesis of Acute Pancreatitis: A Potential Therapeutic Target
Source: Sci Rep. 2017 Apr 6;7:715. doi: 10.1038/s41598-017-00715-6 (PMC5429646; doi:10.1038/s41598-017-00715-6)
Supplement: Supplementary file 1 — Supplementary Materials [file 41598_2017_715_MOESM1_ESM.pdf]

The Role of Heparanase in the Pathogenesis of Acute Pancreatitis: A Potential Therapeutic Target

Iyad Khamaysi[1#*], Preeti Singh[2#], Susan Nasser[3], Hoda Awad[3],

Yehuda Chowers[1], Edmond Sabo[4], Edward Hammond[5], Ian Gralnek[6], Irena Minkov[4],

Alessandro Noseda[7], Neta Ilan[2], Israel Vlodavsky[2] and Zaid Abassi[3,8]

Supplementary Figure Legend

Suppl. Figure 1. A. Heparanase activity. Pancreas tissue was harvested from control WT mice (Con) or WT mice treated with cerulein in the absence (Cer) or presence of Roneparstat (Cer+SST), and pancreatic tissue extracts were evaluated for heparanase activity as described under 'Materials and Methods'. **B**. Immunostaining. Hpa-Tg mice were injected with saline (left panels) or cerulein in the absence (middle panels) or presence (right panels; +SST) of Roneparstat pretreatment. Pancreas tissues were collected 24 h thereafter, and 5 micron sections from formalin-fixed, paraffin-embedded samples were stained for phospho-STAT3 (upper panels) and p65 (lower panels). **C**. Pancreatic index. Pancreatic tissues were collected from control untreated WT (n=13) or Hpa-Tg mice (saline; n=11) or mice treated with cerulein in the absence (cerulein; n=24) or presence of PG545 (cerulein+PG545; n=20) or SST0001 (Cerulein+SST; n=6) and evaluated for pancreatic index (i.e., pancreatic weight/body weight ratio). ***$p<0.01$ for saline vs. cerulein in Hpa-Tg mice; *$p<0.05$ in cerulein vs. cerulein+PG545/SST0001 in WT and Hpa-Tg mice. **D**. IL-10 induction. WT and Hpa-Tg mice were injected with saline (Con) or cerulein in the absence (Cer) or presence of PG545 pretreatment (Cer+PG). Pancreas tissues were collected 24 h thereafter, total RNA was extracted and subjected to real-time PCR analyses applying primers specific for IL-10. **E, F.** Secretion of both lipase and amylase is markedly

inhibited by PG545 administered already 2 h prior to cerulein. Hpa-TG mice were pretreated with PG545 (400 µg/mouse; i.p) or saline administered 24 h (TG+Cerulein+PG early) or 2 h (TG+Cerulein+PG late) prior to induction of AP by cerulein. Lipase (**E**) and amylase (**F**) levels were quantified after 24 h. Note reduced levels of lipase and amylase already upon late administration of PG545.

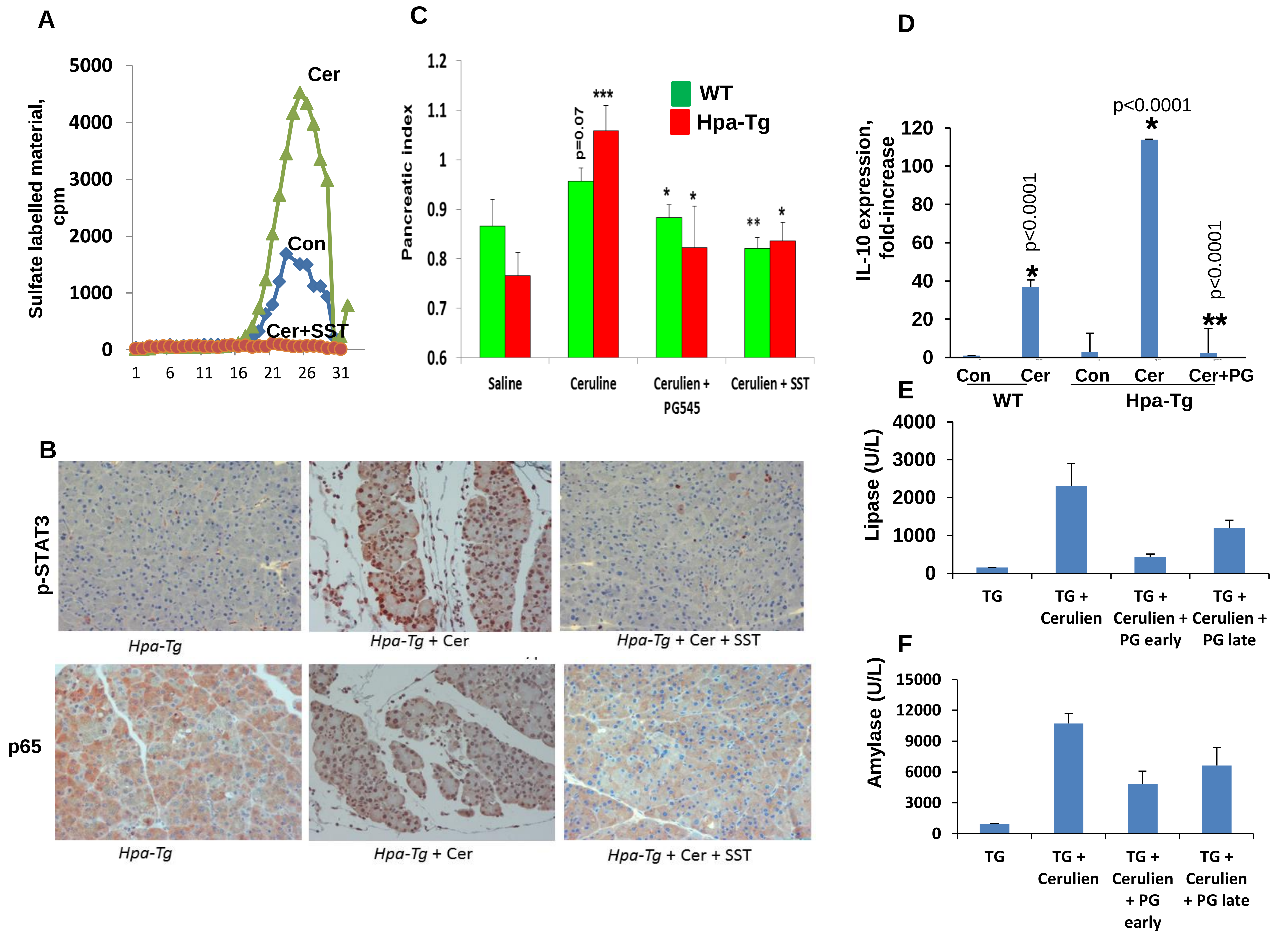
A
Sulfate labelled material, cpm
5000
4000
3000
2000
1000
0
1
6
11
16
21
26
31
Cer
Con
Cer+SST
C
Pancreatic index
1.2
1.1
1
0.9
0.8
0.7
0.6
WT
Hpa-Tg
p=0.07
***
*
*
**
*
Saline
Ceruline
Cerulien + PG545
Cerulien + SST
D
IL-10 expression, fold-increase
120
100
80
60
40
20
0
p<0.0001
*
p<0.0001
*
p<0.0001
**
Con
Cer
Con
Cer
Cer+PG
WT
Hpa-Tg
E
Lipase (U/L)
4000
3000
2000
1000
0
TG
TG + Cerulien
TG + Cerulien + PG early
TG + Cerulien + PG late
F
Amylase (U/L)
15000
12000
9000
6000
3000
0
TG
TG + Cerulien
TG + Cerulien + PG early
TG + Cerulien + PG late
B
p-STAT3
p65
Hpa-Tg
Hpa-Tg + Cer
Hpa-Tg + Cer + SST
Hpa-Tg
Hpa-Tg + Cer
Hpa-Tg + Cer + SST
